# High blood pressure, a red flag for the neonatal manifestation of urea cycle disorders

**DOI:** 10.1186/s13023-019-1055-z

**Published:** 2019-04-08

**Authors:** Ulrike Teufel, Peter Burgard, Jochen Meyburg, Martin Lindner, Johannes Poeschl, Peter Ruef, Georg F. Hoffmann, Stefan Kölker

**Affiliations:** 1 Center for Child and Adolescent Medicine, Division of Pediatric Neurology and Metabolic Medicine, University Hospital Heidelberg, Heidelberg, Germany; 2 Department of General Pediatrics, Adolescent Medicine and Neonatology, Medical Center, Faculty of Medicine, University of Freiburg, Freiburg, Germany; 3 University Children’s Hospital Frankfurt, Frankfurt, Germany; 4 Department of Pediatrics, Clinic of Neonatology, University of Heidelberg, Heidelberg, Germany; 5 Department of Pediatrics, SLK-Kliniken Heilbronn GmbH, Heilbronn, Germany

**Keywords:** Neonatal urea cycle disorders, Hypertension, Clinical presentation, Hyperammonemic encephalopathy

## Abstract

**Background:**

Neonatal manifestation of life-threatening hyperammonemic encephalopathy in urea cycle disorders (UCD) is often misdiagnosed as neonatal sepsis, resulting in significantly delayed start of specific treatment and poor outcome. The major aim of this study was to identify specific initial symptoms or signs to clinically distinguish hyperammonemic encephalopathy in neonates from neonatal sepsis in order to identify affected individuals with UCD and to start metabolic therapy without delay. Furthermore, we evaluated the impact of diagnostic delay, peak plasma ammonium (NH_4_^+^) concentration, mode of emergency treatment and transfer to a tertiary referral center on the outcome.

**Methods:**

Detailed information of 17 patients (born between 1994 and 2012) with confirmed diagnosis of UCD and neonatal hyperammonemic encephalopathy were collected from the original medical records.

**Results:**

The initially suspected diagnosis was neonatal sepsis in all patients, but was not confirmed in any of them. Unlike neonatal sepsis and not previously reported blood pressure increased above the 95th percentile in 13 (81%) of UCD patients before emergency treatment was started. Respiratory alkalosis was found in 11 (65%) of UCD patients, and in 14 (81%) plasma NH_4_^+^concentrations further increased despite initiation of metabolic therapy.

**Conclusion:**

Detection of high blood pressure could be a valuable parameter for distinguishing neonatal sepsis from neonatal manifestation of UCD. Since high blood pressure is not typical for neonatal sepsis, other reasons such as encephalopathy and especially hyperammonemic encephalopathy (caused by e.g. UCD) should be searched for immediately. However, our result that the majority of newborns with UCD initially present with high blood pressure has to be evaluated in larger patient cohorts.

## Background

The physiologic function of the urea cycle is the irreversible fixation of ammonium (NH_4_^+^) to form water-soluble urea and thus to clear excess nitrogen produced by protein catabolism. Deficiency in one of the six enzymes or two transporters of the urea cycle impairs ureagenesis. The majority of these diseases results in hyperammonemia leading to rapidly progressing encephalopathy with severe neurologic sequelae or even death. Patients with the most severe neonatal manifestation present with first symptoms after a short symptom-free interval ranging from a few hours to days, while individuals with attenuated late disease onset may present at any age after the newborn period. The overall prevalence of urea cycle disorders (UCD) has been estimated to be approximately 1 in 35,000 for the United States [1] and 1 in 52,000 live births in Germany, Austria and Switzerland [2]. At least half of them present during the newborn period.

Newborns with UCD initially present with nonspecific symptoms such as vomiting, feeding refusal, irritability, lethargy, respiratory problems and seizures [3–5]. Further progress leads to apnea, cerebral edema and death. Emergency care, stabilization of the neonate during acute illness and immediate start of NH_4_^+^detoxification are indispensable for survival and for the prevention of irreversible brain damage. The clinical phenotype is often mistaken as neonatal sepsis. Since hyperammonemic encephalopathy of UCD patients shares clinical overlap with other diseases becoming manifest in the newborn period, particularly neonatal sepsis, and hence cannot be reliably identified clinically, diagnosis and start of specific emergency therapy is often delayed.

The aim of this study was to identify clinical parameters that help to distinguish between hyperammonemic encephalopathy and neonatal sepsis and reduce diagnostic and therapeutic delay. Furthermore, we evaluated the impact of diagnostic delay, peak plasma ammonium (NH_4_^+^) concentration, mode of emergency treatment and transfer to a tertiary referral center on the outcome.

## Methods

Seventeen patients, born between 1994 and 2012, with confirmed inherited deficiency of argininosuccinate lyase (ASL; MIM #207900), argininosuccinate synthetase 1 (ASS1; MIM #215700), carbamylphosphate synthetase 1 (CPS1; MIM #237300) or ornithine transcarbamylase (OTC; MIM #311250) and neonatal hyperammonemic encephalopathy were included. Sixteen of them were transferred to our center for emergency treatment. Information on pregnancy and delivery, onset of symptoms, diagnostic investigations, treatment protocols and outcome were collected from the original medical records of the transferring hospitals and our center.

IBM SPSS 20 for Windows (SPSS INC., Chicago, IL, USA) was used for statistical analyses. Unless stated otherwise, continuous variables are presented as mean ± standard deviation (SD) and range. The reference values for the age-appropriated 95th percentile of systolic, diastolic and mean arterial blood pressure (MAD) in newborns in the first days of life were based on the values collected by Kent et al. [6, 7]. Differences between groups were tested by Student’s t test or, if normality failed, with Kruskal–Wallis or Mann-Whitney U rank-sum test. *P* values < 0.05 were regarded as statistically significant in a explorative sense.

The study was performed in accordance with the Declaration of Helsinki of 1975, as revised in 2013 after approval by the ethics committee of the University of Heidelberg, Germany (S-416/2011).

## Results

### Study population

Two patients (one female, one male) with CPS1 deficiency, six (all male) with OTC deficiency, seven (three female, four male) with ASS1 deficiency and two (one female, one male) with ASL deficiency were included in this study. All patients except patient #15 (ASS1 deficiency), who was identified by newborn screening, were diagnosed after the manifestation of symptoms during the newborn period.

### Obstetric history and birth

All patients were term newborns, except for patient #6 who was delivered at a gestational age of 36 weeks. Mean gestational age was 39 weeks (SD = 1.5 weeks; range 36 to 41 weeks) and the mean birth weight was 3240 g (SD = 397 g; range 2685 g to 4075 g). Mean APGAR scores were 8.7 (SD = 0.8) at 1 min, 9.9 at 5 (SD = 0.3) and at 10 min (SD = 0.2). Patients were born by vaginal delivery (*n* = 12) or caesarian section (*n* = 5; one primary and four secondary cesarean section).

### Clinical presentation and diagnosis

**Fig. 1:**
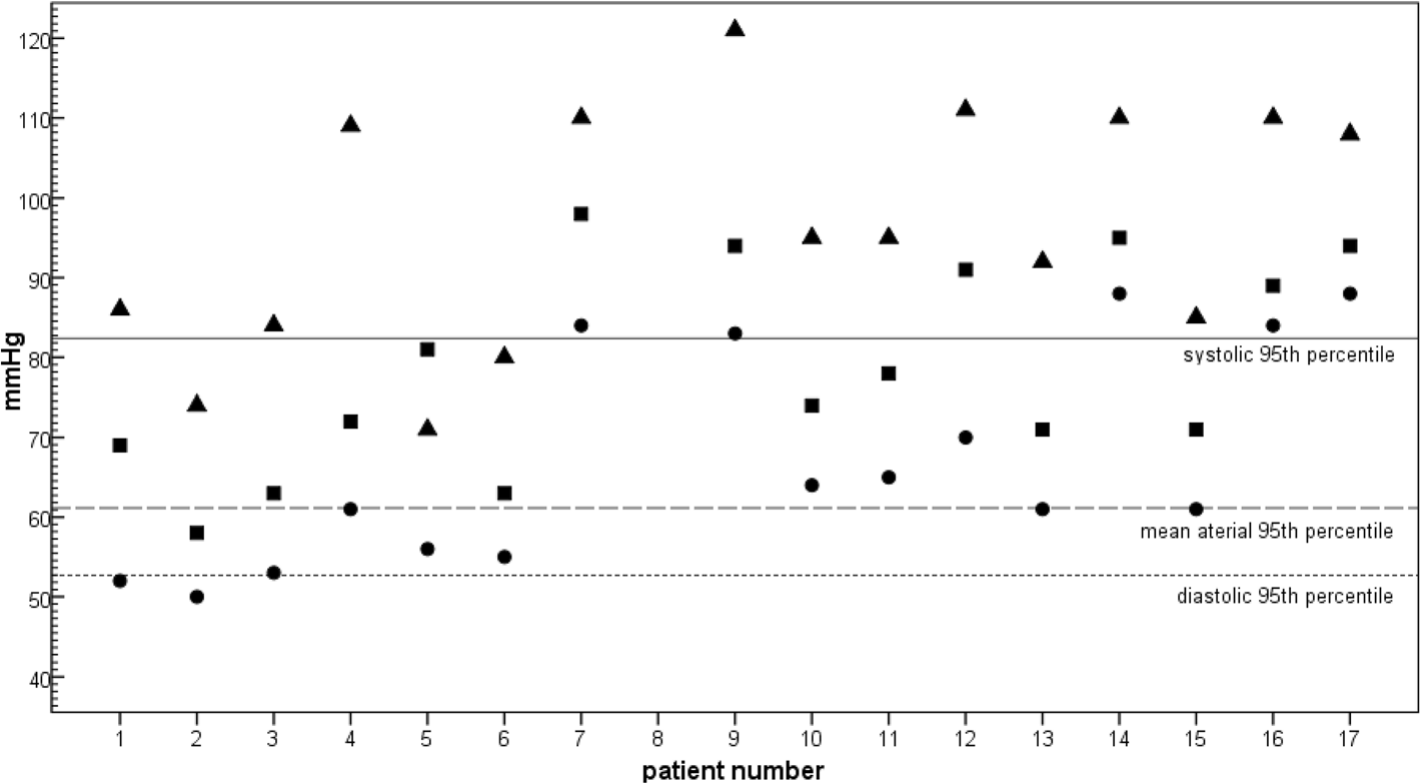
Initial systolic, diastolic and mean arterial blood pressure of the newborns with the respective 95th percentile. Legend: ▲systolic blood pressure; ■ mean aterial blood pressure; ● diastolic blood pressure

Table 1 summarizes initial signs and symptoms of the 17 patients. Mean onset of symptoms was at the 4th day of life (SD = 1.7 days; range 2–9 days). Affected newborns most commonly presented with respiratory distress, muscular hypotonia, and lethargy. Seizures were recognized in one patient before admittance. Interestingly, 13 of 16 patients (81%) presented with increased blood pressure above the 95th percentile before the start of emergency treatment, most of them being lethargic and severely compromised (Fig. 1). Mean systolic, diastolic and MAD pressure was 95 mmHg, 62.5 mmHg, 76 mmHg (SD = 15.3, 13.7, 13.1; range 71–121 mmHg, 50–88 mmHg, 58–98 mmHg). Of note, none of the patients received additional intravenous fluid applications including antibiotic therapy before blood pressure measurements. In the 3 newborns with initial normal blood pressure values, the blood pressure remained at the level and did not increase secondary. Even 3 h after admission to the transferring hospital, 9 of the 13 children with elevated initial blood pressure continued to have elevated blood pressure. Of the remaining four, no blood pressure was documented in three patients at that time and one newborn (#1) was already intubated. Mean systolic, diastolic and MAD pressure was 100 mmHg, 71 mmHg, 86 mmHg (SD = 8.15, 12.7, 10.9; range 89–111 mmHg, 60–94 mmHg, 70–98 mmHg), respectively. Most children experienced a drop in blood pressure with intubation and associated sedation. Looking at the blood pressure in the individual disease groups, an increase from CPS1 via OTC, ASS to ASL was observed for systolic, diastolic and MAD pressure (Table 2). However, there was no significant difference between systolic (*p* = 0.4) and MAD pressure (*p* = 0.21) for each individual disease. Only with diastolic pressure a significance (*p* = 0.038) was detected between CPS1 and ASL. In one patient, initial blood pressure was not reported. In all patients, neonatal sepsis was initially suspected, but was not confirmed in any. With the exception of patient (#1), who was immediately intubated in the referring clinic, none of the newborns initially received a volume bolus.

**Table 1:** Initial clinical presentation and metabolic derangement of neonatal UCD patients

			Initial blood gases				Symptoms^#^						NH_4_^+^			
No	Sex	Deficiency of	pH	Base excess	Lactate	CO_2_	SBP	DBP	MAD	Onset	At obstetric unit	On admission at NICU	First test result	On admission at NICU	Peak NH_4_^+^	Time until NH_4_^+^ < 150 μmol/l
				mmol/l		mmHg				Day of life			(μmol/l)			hours
1°	F	CPS1	7.42	−2.3	6.0	76.7	86	52	69	3	7	1, 3, 6	4000	4420	4420	35
2	M	CPS1	7.36	−9.1	5.7	26.1	74	50	58	3	n.d.	1, 4	673	835	1100	21
3°	M	OTC	7.52	2.9	2.6	29.6	84	53	63	9	4, 8	1, 4, 8	385	136	385	9
4°	M	OTC	7.47	−1.5	4.0	30.0	109	61	72	5	2, 4	2, 4	240	676	676	28
5	M	OTC	7.46	−2.4	5.2	26.0	71	56	81	2	4	1, 4	286	3363	4050	28
6	M	OTC	7.17	−4.1	3.9	n.d.	80	55	63	3	4, 5, 9	1, 4, 6,	1000	1951	2213	30
7°	M	OTC	7.36	1.4	2.5	50.5	110	84	98	4	1	1, 3	1494	3516	3516	34
8	M	OTC	7.48	−4.1	2.9	21.5	n.d.	n.d.	n.d.	3	1	1, 6	750	1750	1750	34
9°	M	ASS1	7.55	0	3.7	25.8	121	83	94	4	1, 2	1, 4	1078	590	1078	12
10°	F	ASS1	7.39	−3.8	7.2	33.2	95	64	74	3	1	1, 3	232	1430	1430	35
11°	M	ASS1	7.55	−5.5	5.2	19.7	95	65	78	4	2,4	1, 4	735	1104	1104	14
12°	F	ASS1	7.55	−2.3	4.9	20.4	111	70	91	4	2, 4, 5	1, 4, 6	484	1091	1091	33
13°	M	ASS1	7.48	−3.4	2.7	24.3	92	61	71	3	1, 6	1, 6	235	1376	1645	24
14°	F	ASS1	7.45	0.4	3.5	34.7	110	88	95	4	1	1	1399	1734	> 2000	15
15^*^†°	M	ASS1	7.34	−7.6	5.9	32.0	85	61	71	7	−/−^*^	4	402	n.d.	402	4
16°	F	ASL	7.36	−4.9	5.8	35.0	110	84	89	3	1, 2, 3	1, 3, 6	1256	1082	1256	24
17°	M	ASL	7.52	2.4	n.d.	29.5	108	88	94	6	1, 2, 4	1, 3, 5	1065	1100	1100	41

*ASL*, Argininosuccinate lyase; *ASS1*, Argininosuccinate synthetase 1; *CPS1*, Carbamylphosphate synthetase 1; *CO*_*2*_, Carbon dioxide; *DBP*, Diastolic blood pressure; *F*, Female; *M*, Male; *NH*_*4*_^*+*^, Plasma ammonium concentration; *No*, Number; *MAD*, Mean arterial pressure; *NICU*, Neonatal intensive care unit; *SBP*, Systolic blood pressure; *OTC*, Ornithine transcarbamylase deficiency;

#Symptoms: 1: respiratory distress, 2: poor feeding, feeding difficulties, 3: muscular hypertonia, 4: lethargy/ muscular hypotonia, 5: hypothermia, 6: acrocyanosis, 7: seizures, 8: vomiting, 9: hypoglycemia

° = blood pressure above the 95th percentile * = identified by newborn screening, †= not transferred to our centre; n.d. = not documented,

**Table 2:** Mean blood pressure, mean initial ammonium level in transferring hospital and at admittance to NICU subdivided by UCD enzyme deficiency

Deficiency of	Blood pressure (mmHg)			NH_4_^+^ (μmol/l)	
	SBP	DBP	MAD	First test result	On Admission at NICU
CPS1	80	51	64	2376	2628
OTC	84	56	72	568	1851
ASS1	95	65	78	484	1240
ASL	109	86	92	1160	1091

*ASL*, Argininosuccinate lyase; *ASS1*, Argininosuccinate synthetase 1; *CPS1*, Carbamylphosphate synthetase 1; *DBP*, Diastolic blood pressure; *NH*_*4*_^*+*^, Plasma ammonium concentration; *No*, Number; *MAD*, Mean arterial pressure; *NICU*, Neonatal intensive care unit; *SBP*, Systolic blood pressure; *OTC*, Ornithine transcarbamylase deficiency;

In the initial work-up, all patients received cranial ultrasound, 14 of them echocardiography and eight abdominal ultrasound. Thorathic X-rays were performed in 13 patients, while eight patients received a lumbar puncture during the work-up of neonatal sepsis. Twelve received intubation and mechanical ventilation due to respiratory distress. One newborn (#1) was intubated immediately after admission to the transferring hospital. The other eleven were intubated before the transfer to our center. Two patients required catecholamines after intubation and eight of them a central venous catheter.

Laboratory tests focusing on parameters of neonatal sepsis (blood count and c-reactive protein (CRP)) were obtained within two hours. Twelve patients received antibiotics immediately after initial blood samples were taken at the transferring pediatric hospital. The antibiotic therapy varied according to the individual standard procedures. Six newborns received antibiotic triple therapy with ampicillin, cephalosporin plus an aminoglycoside. Three newborns obtained ampicillin and aminoglycoside, one received ampicillin plus cephalosporine, one got cephalosporine and glycopeptides and one received only cephalosporine. Blood gases (Table 1) were determined in all patients showing means for pH of 7.46 (SD = 0.1; range 7.17–7.55), base excess (BE) -2.4 (SD = 3.2; range − 9.1 - 2.9) and carbon dioxide (CO_2_) of 29.6 mmHg (SD = 13.6 mmHg; range 19.7–76.7 mmHg). In our population, respiratory alkalosis at initial presentation was present in 11 patients (65%). One patient (#1) with respiratory insufficiency and thus elevated CO_2_ (76.7 mmHg) was intubated immediately after admittance to the referring hospital. Plasma NH_4_^+^ concentrations were determined with a mean delay of 3 h after admission to a Pediatric Department (SD = 11.5 h; range 1–41 h; except for #15 who was identified by newborn screening). Mean initial NH_4_^+^ concentration was 735 μmol/l (SD = 897 μmol/l, range 232–4000 μmol/l). In all patients first therapeutic measures following suspicion of a UCD were transient stop of protein intake and intravenous application of glucose (15–20 g/kg/d, with or without insulin). Two patients (# 1 and #2) received no additional metabolic therapy until in-patient admission in our center. Five patients received intravenous application of only arginine hydrochloride (#7, #8, #10, #14, #18), one patient (#11) only intravenous sodium benzoate and nine patients both medications. Eight patients received L-carnitine.

### Progress after transferal

Overall 16 UCD patients (except #15) from 14 different hospitals were transferred to the neonatal intensive care unit (NICU) of our center after a mean time interval of 1.1 days (SD = 1.1 days; range 0–4 days) following the start of symptoms. Although emergency therapy was started in the referring hospitals, mean plasma NH_4_^+^ concentrations further increased from 735 μmol/l (SD = 897 μmol/l; range 232–4000 μmol/l) to 1240 μmol/l (SD = 1171 μmol/l; range 136–4420 μmol/l) at arrival in our center and three patients required immediate intubation and mechanic ventilation due to respiratory insufficiency. Although intravenous emergency treatment with high glucose, arginine hydrochloride and sodium benzoate was intensified, plasma NH_4_^+^ concentrations increased further in five patients before extracorporeal detoxification was started. Mean maximum NH_4_^+^ concentration was 1537 μmol/l (SD = 1238 μmol/l; range 385–4420 μmol/l). Looking at NH_4_^+^ concentrations in the individual disease groups there was no significant difference between mean initial NH_4_^+^ concentrations (*p* = 0.68) and on admittance at NICU (*p* = 0.74) (Table 2).

Extracorporeal detoxification with hemodialysis/hemofiltration via central venous Shaldon catheter was performed in 15 UCD patients. Mean time interval between admission to our center and start of hemodialysis/hemofiltration was 3.3 h (SD = 1.4 h, range 0.4–5.5. h). A mean duration of 24.8 h (SD 10.4 h, range 4–41 h) was required to reduce plasma NH_4_^+^ concentrations to below 150 μmol/l. Hemodialysis/hemofiltration was continued for a mean of 23.5 h (SD = 14.8 h; range 1.5–52 h). In six patients, plasma NH_4_^+^ concentrations increased again after discontinuation of extracorporeal detoxification. In one patient, hemodialysis/hemofiltration had to be started a second time.

Hypothermia was started in five patients. In analogy to standard protocols for hypothermia treatment in asphyxiated newborns, patients were cooled to 33.5 °C for 72 h using Hico Variotherm 550 (Hirtz). No severe side effects like coagulopathy were observed during hypothermia. After 72 h body temperature was increased by 0.5 °C per hour until a body temperature of 37 °C was reached.

During NICU management three patients required resuscitation. Two patients developed a hypovolemic shock, one (#14) after bleeding from the umbilical vein five days after insertion of the Shaldon catheter, and one (#16) due to high fluid removal during hemodialysis. In the course of resuscitation the latter patient developed a pneumothorax. The third patient (#1) had cardiac arrest following an episode of supraventricular tachycardia.

In patients #5, 7 and 8, therapy was discontinued in the next days to come in agreement with their parents due to an extremely low protein tolerance which was much below minimal requirements for dietary treatment. All of them had severe brain damage which was also confirmed by MRI. They died 7.1 h (mean; SD = 7.1; range 2.25–16.5) after discontinuation of intensive care and metabolic therapy.

Eleven of the surviving patients have been regularly followed by our outpatient clinic until now (mean age: 12 years, SD =5.1 years; range 2.4–19.9). Apart from #3 and #9 all showed impairments of intellectual and motor functions. Both have in common that there was no further increase until the admission in our center, but even a halving of the initial NH_4_^+^ concentration. But all other values such as the time of the first NH_4_^+^ concentration, metabolic emergency therapy, need for ventilation or dialysis differ.

## Discussion

Hyperammonemic encephalopathy in newborns with UCDs is a life-threatening metabolic emergency that requires immediate and targeted treatment to prevent irreversible brain damage and death. The diagnosis of UCDs is often delayed, since clinical presentation shows broad overlap with other neonatal emergencies, in particular neonatal sepsis. The major aim of this study is to better distinguish neonatal onset of UCDs from the more frequent neonatal sepsis. The clinical presentation of UCD patients in this study is in line with previous reports on neonatal onset of UCD patients [3–5]: Progressive respiratory distress, muscular hypotonia and lethargy were the most frequent clinical symptoms. Striking and not previously reported was the finding of high blood pressure during hyperammonemic encephalopathy, a finding clearly distinguishing between UCDs and neonatal sepsis. Blood pressure was determined by oscillometry. Even in pediatric population oscillometric and radial artery blood pressure are closely correlated with only a small error [8]. In contrast to preterm infants there is no correlation between blood pressure and birth weight, length or gestational age in term infants [6, 7, 9]. In our cohort except one all were term infants. As described above 13 out of 16 patients (81%) in our study had a blood pressure above the age-appropriate 95th percentile [6] at first contact with a pediatrician due to clinical worsening, with most neonates being lethargic and severely compromised. Therefore high blood pressure was not due to increased activity or additional fluid administration. Beside activity an alternative cause of neonatal hypertension is increased intracranial pressure due to cerebral edema [10]. The initial cranial ultrasound including Doppler and Power color Doppler examination, however, showed no evidence for an apparent brain edema on admittance. Alternatively, low plasma arginine concentrations, which are characteristic for urea cycle disorders, can also be a reason for arterial hypertension. Production of arginine is important for endogenous cellular production of nitric oxide (NO), a potent vasodilator [11]. Patients with UCD, except those with arginase 1 deficiency, have low arginine levels. In ASL deficiency impaired systemic NO production is thought to rely on disturbed formation of a complex including ASL and endothelial NO synthase among other components [12]. In a newborn receiving arginine for evaluation of pituitary function a drop of blood pressure was seen after infusion of arginine followed by re-rising after discontinuation [13]. Nelin et al. [14] showed that arginine infusion lowers blood pressure in normotensive infants. A few cases reported about children with ASL deficiency and arterial hypertension. Arterial hypertension was resolved with intravenous infusion of arginine [13, 15]. We aimed to test this hypothesis in our study population, however, plasma arginine concentrations were determined only in 5 of the 17 patients before starting metabolic emergency treatment which also includes arginine. In addition, we were able to show that the initial high blood pressure is not a one-off event, but initially persisted in newborns with UCD. For most of them, sedation and intubation led to a drop in blood pressure. Since in neonatal sepsis and other causes of severe disease systemic blood pressure is mostly reduced [16], elevated blood pressure, probably readily explained by the pathophysiology of UCD, may be an important indicator for UCD.

Blood gases are often the first quantitative data available in a very sick infant. Since respiratory alkalosis was present in 65% of patients at initial presentation, this finding should be considered as another indicator of UCDs in any rapidly deteriorating neonate [5, 17].

Three out of the 17 patients died in the course of their first crisis. All others stayed alive, and 11 patients are still followed regularly in our outpatient clinics (mean follow-up time 13.6 years, SD = 5.5 years). Only two of the 11 surviving patients (#3: peak plasma NH_4_^+^ concentrations: 590 μmol/l and #9: 385 μmol/l) are not cognitively disabled. Their favorable neurological outcome may be due to only moderate peak plasma NH_4_^+^ concentrations during the neonatal crisis. In addition to age at disease onset the initial peak plasma NH_4_^+^ concentration is an important predictor of the neurological outcome [18, 19]. In a cohort of 456 patients, Posset et al. [18] described an initial peak NH_4_^+^above 500 μmol/l to be associated with neurological impairment. In 88 patients with UCDs reported by Bachmann et al. [19] none of the patients with an initial NH_4_^+^ level above 300 μmol/l (first test result) or 480 μmol/l (peak) achieved a normal neurological outcome. This has been confirmed by other studies [20, 21]. In our population we noticed no significant differences of plasma NH_4_^+^ concentrations between specific UCDs, whereby number of patients was small. Early diagnosis and immediate start of metabolic therapy aiming to normalize plasma NH_4_^+^concentrations is the prerequisite to prevent irreversible brain damage [22]. Noteworthy, until extracorporeal detoxification was started, plasma NH_4_^+^ concentrations further increased in the majority of patients despite intensified intravenous therapy with sodium benzoate, arginine and high-dose glucose therapy in combination with insulin. This highlights that intravenous emergency treatment is often insufficient to lower plasma NH_4_^+^ concentrations rapidly and reliably and therefore hemodialysis has to be considered early. From this it can be concluded that a timely transport of every patient with hyperammonemic encephalopathy to a metabolic center which operates hemodialysis in neonates should be considered, planned and organized.

Five patients were treated with hypothermia in addition to pharmacologic and extracorporeal detoxification. This potentially neuroprotective intervention has been described in animal experiments and small clinical trials in hepatic encephalopathy. One of the protective effects of hypothermia may be the prevention of osmolyte depletion in the brain. In astrocytes, NH_4_^+^is reversibly fixed to glutamate forming. The NH_4_^+^/glutamine/brain swelling hypothesis of hepatic encephalopathy suggests that accumulation of glutamine causes astrocytes to swell [23]. Decreased NH_4_^+^ delivery to the brain when patients with acute liver failure were cooled was referred to by Jalan [24, 25]. Polderman [26] reported a reduction of the metabolic rate by 8% per degree Celsius. There is one study reporting first results about feasibility of therapeutic hypothermia in neonatal hyperammonemia due to UCD [27].

## Conclusion

Unfortunately the diagnosis of UCDs is often delayed [28]. Therefore it is most important to further increase the awareness for UCDs as a differential diagnosis in acutely ill neonates. Until now respiratory alkalosis had been recognized as an easily detectable, but inconsistent initial finding of UCDs [29]. Our study confirms this previous observation and highlights for the first time that elevated blood pressure might be an even better indicator of UCDs in newborns and distinguishes it from more frequent neonatal sepsis. Blood gases, blood pressure and plasma NH_4_^+^ should be determined without delay in every sick neonate with suspected neonatal sepsis.

## Abbreviations

ASL: Argininosuccinate lyase deficiency
ASS1: Argininosuccinate synthetase 1 deficiency
BE: Base excess
CO_2_: Carbon dioxide
CPS1: Carbamylphosphate synthetase 1 deficiency
CRP: C-reactive protein
e.g: for example
g: gram
MAD: mean arterial blood pressure
mg: milligram
MRI: magnetic resonance imaging
n: number
NH_4_^+^: ammonium
NICU: Neonatal intensive care unit
NO: Nitric oxide
OTC: Ornithine transcarbamylase deficiency
SD: Standard deviation
UCD: urea cycle disorders
